# All-Trans Retinoic Acid Improves the Effects of Bone Marrow-Derived Mesenchymal Stem Cells on the Treatment of Ankylosing Spondylitis: An *In Vitro* Study

**DOI:** 10.1155/2015/484528

**Published:** 2015-06-01

**Authors:** Deng Li, Peng Wang, Yuxi Li, Zhongyu Xie, Le Wang, Hongjun Su, Wen Deng, Yanfeng Wu, Huiyong Shen

**Affiliations:** ^1^Department of Orthopedics, Sun Yat-sen Memorial Hospital, Sun Yat-sen University, Guangzhou 510120, China; ^2^Center for Biotherapy, Sun Yat-sen Memorial Hospital, Sun Yat-sen University, Guangzhou 510120, China

## Abstract

Previous studies have demonstrated the immunosuppressive effects of both all-trans retinoic acid (ATRA) and mesenchymal stem cells (MSCs). The present study aimed to assess the immunoregulatory effects of ATRA on MSCs in the treatment of ankylosing spondylitis (AS). Bone marrow-derived MSCs from healthy donors were pretreated with ATRA and cocultured with CD3/28-activated peripheral blood mononuclear cells (PBMCs) derived from AS patients. Frequencies of Th17 and regulatory T (Treg) cells were analyzed using flow cytometry. The secretion and the mRNA level of key cytokines were measured with cytometric bead array and quantitative real-time PCR, respectively. ATRA pretreatment increased interleukin-6 (IL-6) secretion of MSCs. Th17 and Treg subset populations were increased and reduced by ATRA-pretreated MSCs, respectively. ATRA-pretreated MSCs significantly decreased not only the vital pathogenic cytokine in AS, tumor necrosis factor-*α* (TNF-*α*), but also AS-boosting factors interleukin-17 (IL-17A) and interferon-*γ* (IFN-*γ*). These results indicated that IL-6 may be a potential protective factor in AS and highlighted the promising role of ATRA in improving the efficacy of MSC-based treatment of AS.

## 1. Introduction

Ankylosing spondylitis (AS) is a chronic, progressive inflammatory disease affecting primarily the axial skeleton. AS is considered as an autoimmune disease and interleukin-17A (IL-17A-) producing Th17 cells are involved in AS pathogenesis [1, 2].

For the first time, we have reported that allogenic intravenous infusion of bone marrow-derived mesenchymal stem cells was an effective and safe method for the treatment of AS, especially cases for which etanercept and NSAIDs are ineffective [3]. However, the underlying mechanisms remain largely unknown. Mesenchymal stem cells (MSCs) are multipotent progenitor cells, which exhibited immunomodulatory capacity [4] and low immunogenicity [5]. MSCs have been successfully used in the treatment of a number of autoimmune/inflammatory diseases such as Crohn's disease [6] and systemic lupus erythematosus [7] on the base of their immunosuppressive ability. The mechanisms of immunomodulation are quite complicated and still not completely clear. Some studies demonstrated that cell-cell contact is involved in the immunosuppressive action, such as inhibition of Th17 differentiation and IL-17 secretion [8, 9]. MSCs can also attract effector T cells through secreting chemokines to facilitate direct immunomodulation [10] or trigger FAS/FASL-induced apoptosis [11]. Parallel to this, MSC secretome is also reported contributing to cell-contact independent effects. The most studied soluble molecules derived from MSCs include indoleamine 2,3-dioxygenase (IDO), prostaglandin E2 (PGE2), transforming growth factor-beta (TGF-*β*), nitric oxide (NO), and hepatocyte growth factor (HGF) [10, 12].

All-trans retinoic acid (ATRA), a representative metabolite of retinol, is involved in a number of biological activities of retinol. ATRA plays a key role in cell proliferation, differentiation, and maturation [13]. In addition, ATRA has been shown to regulate innate immunity through inducing regulatory T cells (Tregs) and suppressing Th17 differentiation [14]. Several studies have documented the efficacy of ATRA in the treatment of autoimmune diseases [15–17]. The retinol level in AS patients was significantly lower than that in healthy controls [18]. It has also been observed that ATRA administration reduced the frequency of Th17 and inhibited the secretion of TNF-*α* in peripheral blood of AS patients [19].

Taken together, we hypothesize that ATRA is effective for the treatment of AS through regulating the immunomodulatory capacity of MSCs. In the present study, we cocultured MSCs and peripheral blood mononuclear cells (PBMCs) and examined the frequencies of Treg and Th17 subsets and the secretion of key cytokines. We defined the pathogenic Th17 cells with a combination of the surface markers of CD4, CCR4, and CCR6 [20]. To the best of our knowledge, this is the first study showing that ATRA improved the immunomodulatory ability of MSCs for the treatment of AS.

## 2. Materials and Methods

### 2.1. Isolation of MSCs and PBMCs

This study was approved by the Ethics Committee of Sun Yat-sen University, Guangzhou, China. Healthy volunteers (*n* = 20, 15 males, 5 females, age, 18–28 years) were recruited as donors of bone marrow. Bone marrow was extracted from the posterior superior iliac spine under a sterile condition and MSCs were isolated according to standardized procedures [21]. MSCs of the fourth passage were used in experiments. PBMCs were isolated from the peripheral blood of AS patients (*n* = 24, 18 males, 6 females, age, 16–40 years) using Ficoll-Hypaque gradient centrifugation. Patients were selected according to modified New York criteria [22]. Informed consent was obtained from all donors and patients.

### 2.2. Trilineage Differentiation Potential Assays of MSCs

*Osteogenic Differentiation.* MSCs were seeded into six-well plates (1 × 10^5^ cells/well) in a total volume of 3 mL DMEM medium supplemented with 10% fetal bovine serum (FBS), 50 mg/L ascorbic acid (Sigma), 10 mM *β*-glycerophosphate (Sigma), and 100 nM dexamethasone (Sigma). The medium was replaced every three days. Total culture duration was 21 days. Alizarin red staining was used.

*Chondrogenic Differentiation.* MSCs (2.5 × 10^5^ cells) were centrifuged at 600 g for 5 minutes in a 15 mL conical tube. Cells were cultured with high-glucose DMEM supplemented with 1% ITS-Premix (Corning), 50 mg/L ascorbic acid (Sigma), 1 mM sodium pyruvate (Sigma), 100 nM dexamethasone (Sigma), and 10 ng/mL transforming growth factor-*β*3 (R&D). The medium was replaced every three days. Total culture duration was 21 days.

*Adipogenic Differentiation.* MSCs were seeded into six-well plates (1 × 10^5^ cells/well). DMEM medium was supplemented with 10% FBS, 1 *μ*M dexamethasone (Sigma), 10 *μ*g/mL insulin (Sigma), 0.5 mM 3-isobutyl-1-methylxanthine (Sigma), and 0.2 mM indomethacin (Sigma). After 3 days' induction, the medium was replaced with DMEM containing 10 *μ*g/mL insulin, and 1 day later, the medium was replaced with the inducing medium mentioned above. After three cycles of such culture, cells were coated with DMEM containing 10 *μ*g/mL insulin until 14th day. Oil red O staining was used after paraformaldehyde fixation.

### 2.3. ATRA Preparation and MSCs Pretreatment

ATRA powder (Sigma) was dissolved in dimethyl sulfoxide (DMSO) and stocked according to the manufacturer's instructions. ATRA solution was prepared with DMEM medium supplemented with 10% FBS. MSCs were seeded into six-well plates (1 × 10^5^ cells/well) in a total volume of 3 mL DMEM medium, pretreated by 1 *μ*M ATRA for 1 day, 3 days, and 5 days according to different experiments. The MSCs cocultured with PBMCs were only pretreated for 1 day. After pretreatment, the medium containing ATRA was completely removed and MSCs were washed with phosphate buffer saline (PBS) for three times. Cells were then cultured in ATRA-free medium. MSCs of the control group were pretreated with DMSO of equal volume to ATRA.

### 2.4. Cell Culture

MSCs were cocultured with PBMCs at a ratio of 1 : 20 of MSC : PBMC. T cells were stimulated by purified anti-CD3 (0.2 *μ*g/mL, BD Pharmingen) and anti-CD28 (1 *μ*g/mL, BD Pharmingen) antibodies. Cells were cocultured in RPMI-1640 medium of an ultimate volume of 3 mL for 5 days. The cell ratio and the coculture duration were determined according to preliminary experiments. For CD4+ T cell proliferation analysis, PBMCs were incubated with 5 *μ*M carboxyfluorescein diacetatesuccinimidyl ester (CFSE, Life Technologies) for 15 minutes and washed with PBS containing 10% FBS three times before coculture. To evaluate the effects of proinflammatory cytokines on IL-6 secretion of MSCs, exogenous interferon-*γ* (IFN-*γ*, PeproTech) (10 ng/mL) and TNF-*α* (10 ng/mL, R&D) were added into the culture medium of pretreated MSCs which were cultured for the following 5 days. For cytokine measurement, a noncontact coculture was also conducted using a six-well and 0.4 *μ*m pore Transwell plate system (Corning) in which PBMCs were seeded in the upper chamber.

### 2.5. Flow Cytometry

MSCs were trypsinized for identification of a number of surface markers. The antibodies for surface markers were anti-CD29-PE, anti-CD34-APC, anti-CD44-FITC, anti-CD45-FITC, anti-CD90-PE, anti-CD105-FITC, and anti-HLA-DR-PE (all from BD Pharmingen). PBMCs were harvested through centrifugation at the end of coculture. For proliferation analysis of CD4+ T cells, CFSE-incubated PBMCs were marked with anti-CD4-PE (BD Pharmingen), cells left-shifting into the square gate were recognized as the proliferating cells, and the cell count percentage within this gate represented the proliferation rate. For Th17 analysis, anti-CD4-FITC, anti-CCR4-PerCP-Cy5.5, and anti-CCR6-APC (all from BD Pharmingen) were used. The human regulatory T cell staining kit (eBioscience) containing anti-CD4-FITC/CD25-APC cocktail and anti-Foxp3-PE was used in Treg analysis according to the manufacturer's instruction. Cells were measured in a flow cytometry system (BD FACSVerse).

### 2.6. Cytometric Bead Array (CBA)

Culture supernatant was collected for cytokine measurement using a CBA kit (Human Th1/Th2/Th17 Kit, BD Pharmingen) according to the manufacturer's instruction. Cytokine concentrations were presented as PE fluorescence intensities relative to a standard curve.

### 2.7. Quantitative Real-Time PCR (qPCR)

Total RNA was extracted using the TRIzol reagent (Life Technologies). Synthesis of cDNA was performed using a PrimeScript RT reagent kit (Takara) according to manufacturer's instructions. The qPCR was performed on a LightCycler 480 Real-Time PCR System (Roche) using SYBR Premix Ex Taq (Takara). The PCR program was 30 s at 95°C, 40 cycles of 5 s at 95°C, and 20 s at 60°C. Relative expression changes in mRNA levels of genes were assessed using 2^(−ΔΔct)^ method and normalized to the housekeeping gene* GAPDH*. The sequences of primers used in the qPCR assay were as follows:* IL-6*: 5′-CCTGAACCTTCCAAAGATGGC-3′ (forward), 5′-TTCACCAGGCAAGTCTCCTCA-3′ (reverse);* IL-17A*: 5′-TCCCACGAAATCCAGGATGC-3′ (forward), 5′-GGATGTTCAGGTTGACCATCAC-3′ (reverse);* TNF-α*: 5′-CCTCTCTCTAATCAGCCCTCTG-3′ (forward), 5′-GAGGACCTGGGAGTAGATGAG-3′ (reverse);* IFN-γ*: 5′-TCGGTAACTGACTTGAATGTCCA-3′ (forward), 5′-TCGCTTCCCTGTTTTAGCTGC-3′ (reverse);* GAPDH*: 5′-GGAGCGAGATCCCTCCAAAAT-3′ (forward), 5′-GGCTGTTGTCATACTTCTCATGG-3′ (reverse).

### 2.8. Statistical Analyses

Data were presented as mean ± SD. One-way ANOVA or *t*-test was used according to the type of data. Statistical analyses were conducted using SPSS software (SPSS Inc.). A *P* value less than 0.05 was considered to be significantly different.

## 3. Results

### 3.1. Phenotypic Characterization and Trilineage Differentiation of MSCs

**Figure 1:**
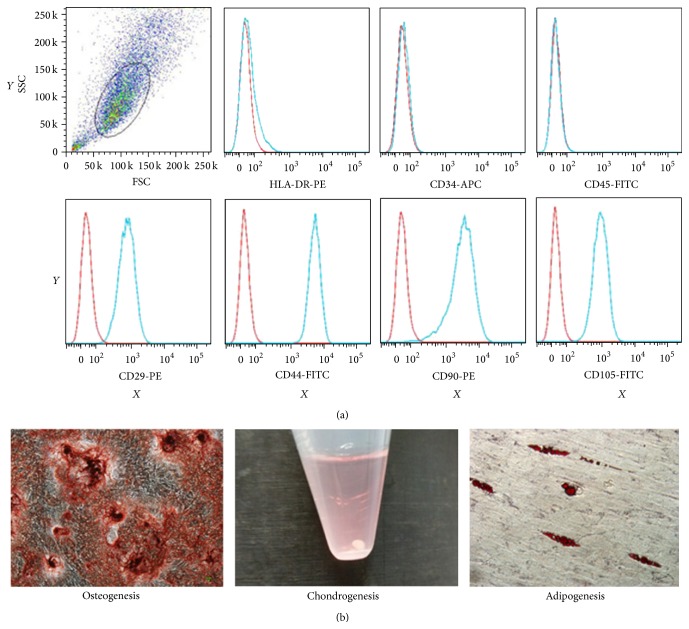
Identification of MSCs. (a) Phenotypic characterization based on a number of surface markers. MSCs were positive for CD29, CD44, CD90, and CD105, but negative for HLA-DR, CD34, and CD45. Red line: background fluorescence of isotype controls. Blue line: fluorescence intensity from selected markers. *X*-axis: fluorescence intensity. *Y*-axis: cell count. (b) Trilineage differentiation.

Flow cytometric analysis was used to identify phenotypic surface markers of MSCs. All MSCs from different donors were positive of CD29, CD44, CD90, and CD105 but negative of CD34, CD45, and HLA-DR, confirming the typical MSC phenotypes (Figure 1(a)). Osteogenic, chondrogenic, and adipogenic differentiations were successfully induced (Figure 1(b)).

### 3.2. MSCs Inhibited the Proliferation of CD4+ T Cells and ATRA Reduced This Inhibitory Ability

**Figure 2:**
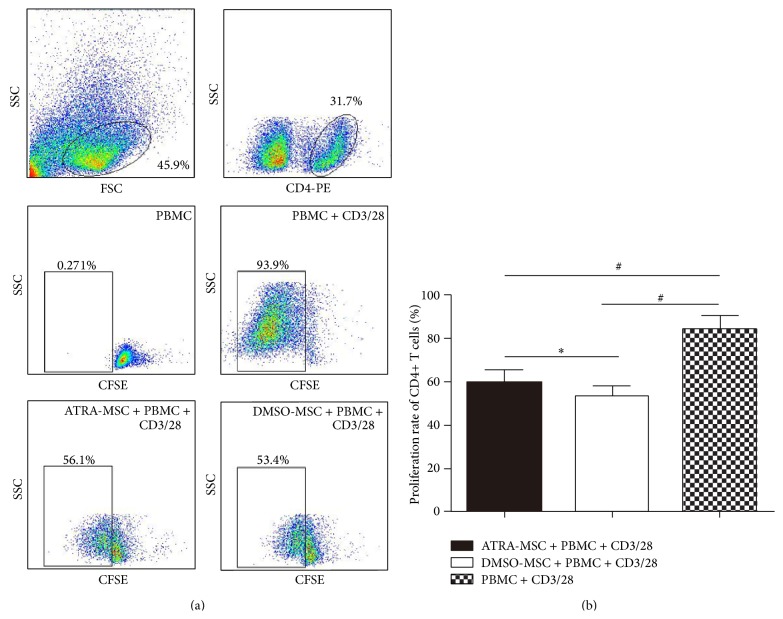
The proliferation rate of CD4+ T lymphocytes incubated with CFSE. (a) Nonactivated T cells were used as controls. (b) When stimulated by CD3/28, the proliferation rate of CD4+ T cells was extremely high (83.7 ± 6.7%). When cocultured with MSCs, the proliferation of CD3/28-stimulated CD4+ T cells was inhibited, but a slightly higher proliferation rate was observed in ATRA-pretreated MSCs (58.8 ± 6.2%) than that in DMSO-pretreated MSCs (52.4 ± 5.5%). One-way ANOVA was used. ^∗^ *P* < 0.05 and ^#^ *P* < 0.001.

The proliferation of CD4+ T cells was measured according to fluorescence intensities. The 0 day PBMCs (noncocultured, not activated by CD3/28) group was defined as the nonproliferation control. When stimulated by CD3/28, the proliferation rate of CD4+ T cells was extremely high (83.7 ± 6.7%), suggesting an efficient proliferation. When cocultured with MSCs, the proliferation of CD3/28-stimulated CD4+ T cells was inhibited (*P* < 0.001), but a slightly higher proliferation rate was observed in ATRA-pretreated group (58.8 ± 6.2%) than that in DMSO-pretreated group (52.4 ± 5.5%) (*P* < 0.05). Thus, the ability inhibiting the proliferation of CD4+ T cells of ATRA-pretreated MSCs was lower than control (Figure 2).

### 3.3. ATRA-Pretreated MSCs Reduced the Treg but Increased the Th17 Subpopulation

**Figure 3:**
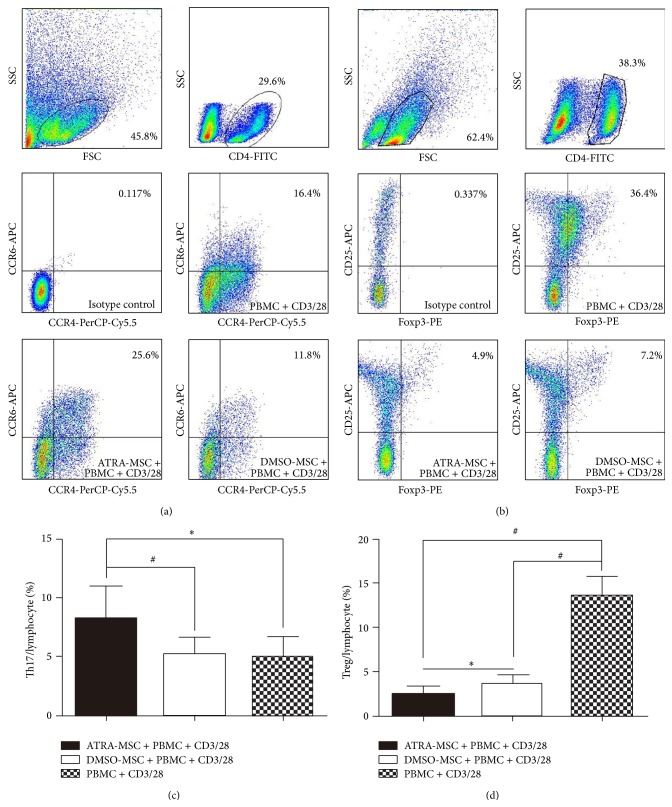
Effects of ATRA-pretreated MSCs on Th17 and Treg subpopulations. (a and c) The percentage of CD3/28-stimulated Th17 subset (Th17/lymphocyte). (b and d) The percentage of CD3/28-stimulated Treg subset (Treg/lymphocyte). One-way ANOVA was used. ^∗^ *P* < 0.05 and ^#^ *P* < 0.001.

The percentage of CD3/28-stimulated Th17 subset (Th17/lymphocyte) was increased from 4.9 ± 1.8% to 8.2 ± 2.7% (*P* < 0.05) by ATRA-pretreated MSCs. ATRA pretreatment led to an expansion of Th17 subset (Figures 3(a) and 3(c)), but the control DMSO pretreatment did not cause a Th17 expansion compared with the noncocultured group. The percentage of CD3/28-stimulated Treg subset (Treg/lymphocyte) was decreased from 13.7 ± 2.1% to 2.5 ± 0.9% (*P* < 0.001) and 3.6 ± 1.0% (*P* < 0.001) by ATRA-pretreated MSCs and DMSO-pretreated MSCs, respectively. Thus, MSCs caused a significant decrease in the Treg subpopulation, and ATRA pretreatment further reduced the Treg subset (*P* < 0.05) (Figures 3(b) and 3(d)).

### 3.4. The Secretion and mRNA Level of Interleukin-6 (IL-6) Was Increased by ATRA Pretreatment

**Figure 4:**
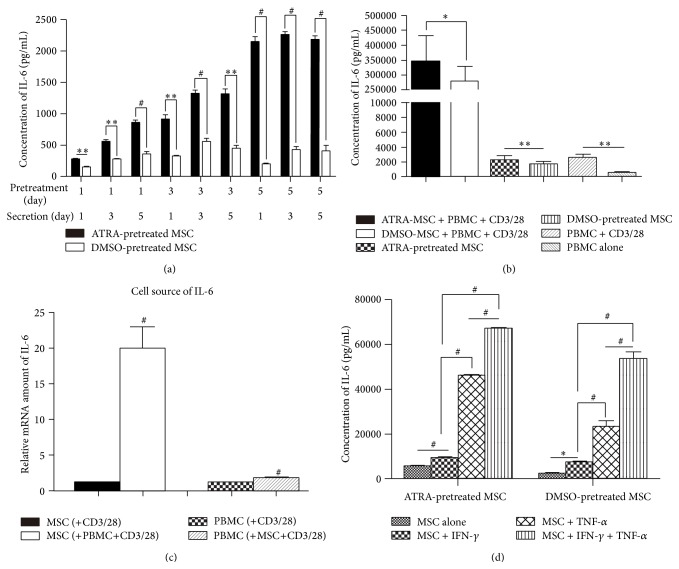
Changes of IL-6 levels under different conditions. (a) Concentration of IL-6 secreted by MSCs pretreated for different durations (1 day, 3 days, and 5 days). The secreting durations (1 day, 3 days, and 5 days) after pretreatment were different, too. Paired *t*-test was used. (b) Concentration of IL-6 in coculture and noncoculture groups. Paired *t*-test was used. (c) Relative mRNA amount showed the principal cell source of IL-6. (d) Effects of IFN-*γ* and TNF-*α* on production of IL-6 by MSCs. One-way ANOVA was used. ^∗^ *P* < 0.05, ^∗∗^ *P* < 0.01, and ^#^ *P* < 0.001.

We found MSCs constitutively secreted only IL-6 but none of other cytokines within the spectrum of CBA kit, and the secretion of IL-6 was increased by ATRA pretreatment in an approximately time-dependent manner, which was not seen in DMSO-pretreated groups (Figure 4(a)). In the coculture of MSCs and PBMCs, the IL-6 level in the supernatant was significantly increased by over 150-fold at average compared with the IL-6 level in MSCs or PBMCs cultured alone and was even higher in ATRA-pretreated cultures than relative DMSO-pretreated control groups (*P* < 0.05) (Figure 4(b)). These findings were found in both contact and noncontact cocultures. The mRNA level of IL-6 was not significantly changed in PBMCs, but it increased dramatically in MSCs, suggesting that IL-6 induced in the coculture of MSCs and PBMCs was mainly secreted by MSCs rather than PBMCs (Figure 4(c)).

### 3.5. TNF-*α* and IFN-*γ* Increased IL-6 Production by MSCs

Exogenous TNF-*α* and IFN-*γ* added into the culture medium of pretreated MSCs significantly and synergistically enhanced the IL-6 secretion of MSCs. TNF-*α* was more efficient than IFN-*γ* in upregulating IL-6 production with an equal dose (*P* < 0.001). Just like the results presented earlier, ATRA-pretreated MSCs produced higher level of IL-6 than MSCs without ATRA stimulation (Figure 4(d)).

### 3.6. Changes of Cytokines of IL-17A, IFN-*γ*, and TNF-*α* by ATRA Pretreatment

**Figure 5:**
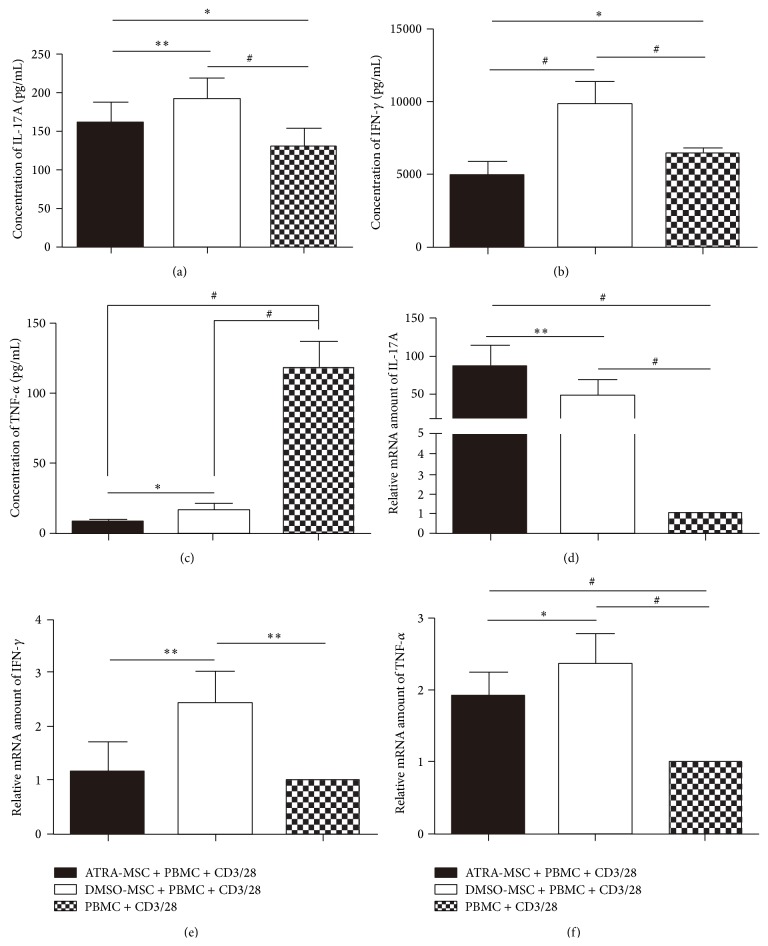
Secretion and gene transcription of IL-17A, IFN-*γ*, and TNF-*α* in the coculture of MSCs and PBMCs. (a) Results of IL-17A secretion from CBA. (b) Results of IFN-*γ* secretion from CBA. (c) Results of TNF-*α* secretion from CBA. (d) Results of IL-17A gene transcription from qPCR. (e) Results of IFN-*γ* gene transcription from qPCR. (f) Results of TNF-*α* gene transcription from qPCR. One-way ANOVA was used. ^∗^ *P* < 0.05, ^∗∗^ *P* < 0.01, and ^#^ *P* < 0.001.

MSCs increased IL-17A production, which was lower in the ATRA-pretreated group (161.2 ± 26.0 pg/mL) compared with the DMSO-pretreated group (191.0 ± 27.3 pg/mL) (*P* < 0.01) (Figure 5(a)). IFN-*γ* was increased from 6420.2 ± 374.0 pg/mL to 9844.9 ± 1553.0 pg/mL (*P* < 0.001) by DMSO-pretreated MSCs but decreased to 4920.2 ± 977.8 pg/mL (*P* < 0.05) by ATRA-pretreated MSCs (Figure 5(b)). The TNF-*α* level was significantly reduced from 118.5 ± 17.8 pg/mL to 8.3 ± 1.8 pg/mL (*P* < 0.001) and 16.8 ± 4.1 pg/mL (*P* < 0.001) by ATRA-pretreated MSCs and DMSO-pretreated MSCs, respectively (Figure 5(c)). These findings were found in both contact and noncontact cocultures.

### 3.7. Gene Transcription of IL-17A, IFN-*γ*, and TNF-*α*

The variations of the mRNA levels of IL-17A, IFN-*γ*, and TNF-*α* from PBMCs were also measured. MSCs significantly increased IL-17A gene transcription, especially in ATRA pretreatment group (*P* < 0.001) (Figure 5(d)). MSCs increased the mRNA level of IFN-*γ*, which was lower in the ATRA-pretreated group compared with the DMSO-pretreated group (*P* < 0.01) (Figure 5(e)). MSCs unexpectedly increased the gene transcription of TNF-*α* even in ATRA pretreatment group (*P* < 0.001) (Figure 5(f)). These findings were found in both contact and noncontact cocultures.

## 4. Discussion

CD4+ T cells are also known as helper T cells. Our results showed that MSCs inhibited CD4+ T cell proliferation, and the inhibitory ability of ATRA-pretreated MSCs was slightly weaker than that of controlled DMSO-pretreated MSCs. Therefore, ATRA could probably affect the immune nature of MSCs by regulating the proportions of T helper effector cells.

We initially expected an increase of Treg subset and a decrease of Th17 subset populations after MSCs were cocultured with PBMCs. However, we observed opposite results. Briefly, MSCs caused a decline of Treg subset and a slight increase of Th17 subset populations. Similar findings have been reported by Guo et al. [23]. Interestingly, ATRA pretreatment further augmented these unexpected changes. We noted that ATRA pretreatment increased the secretion of IL-6 by MSCs and the level of IL-6 correlated with Th17 subpopulation. It has been confirmed that IL-6 inhibited the generation of Tregs induced by TGF-*β* and caused* T*_0_ skews for Th17 [24]. Therefore, the Th17-skewing triggered by coculture was probably regulated by increased production of IL-6.

IL-6 has long been recognized as a proinflammatory cytokine and a therapeutic target, because IL-6, together with TNF-*α* and IL-1, was upregulated in most inflammatory conditions [25]. Some studies suggested that blocking IL-6 was an efficient way for the treatment of AS [26, 27]. However, the most recent randomized, placebo-controlled trials demonstrated that blocking IL-6 with antibodies against IL-6 receptor-*α* was not effective for the treatment of AS [28, 29]. Furthermore, no axial improvements were observed in AS patients treated with tocilizumab, an antibody against IL-6 receptor, in a multiple center study [30]. Currently, no IL-6 blocker is approved for the treatment of AS. The level of IL-6 produced by MSCs was increased by over 150-fold when MSCs were cocultured with activated PBMCs according to our results and we demonstrated the upregulation of IL-6 was cell-contact independent. Therefore, we intended to figure out the factors responsible. We chose TNF-*α* and IFN-*γ* for investigation. Firstly, both TNF-*α* and IFN-*γ* were classical proinflammatory cytokines and were elevated in various inflammatory statuses [31]. Secondly, cell-free macrophage conditioned medium significantly increased the secretion of IL-6 of MSCs [32], while TNF-*α* was the primary production of macrophage. Thirdly, IFN-*γ* was confirmed to be the boost signal for MSC licensing related to immunomodulation of MSCs [33]. Our data showed that TNF-*α* and IFN-*γ* significantly and synergistically enhanced IL-6 secretion of MSCs, suggesting that the upregulation of IL-6 depends probably on environmental mediators and may be associated with the immunomodulation of MSCs. These observations raised a question on whether IL-6 is a proinflammatory or an anti-inflammatory cytokine in AS. The increased production of IL-6 by MSCs would have aggravated the damage, if IL-6 was proinflammatory in AS. But according to our previous study, MSC infusion was safe and effective for the treatment of AS [3]. In addition, ATRA pretreatment caused a subsequent decline of TNF-*α*, IL-17A, and IFN-*γ*, the recognized pathogenic cytokines in AS. Taken together, we speculate that IL-6 secreted by MSCs is a reactive rather than an initiative cytokine responding to inflammatory environment, and IL-6 may be a protective agent rather than a pathogenic factor in the pathogenesis of AS. In fact, some studies did indicate that IL-6 displayed anti-inflammatory nature [34–36].

Higher frequency of peripheral Th17 and IL-17A has been detected in AS patients compared to healthy controls [1, 2]. In the present study, we found that IL-17A production was increased by MSCs. Our results also revealed a positive association between the mRNA level of IL-17A and the Th17 subpopulation. However, we observed a reduction in IL-17A secretion in the ATRA-pretreated group compared with the DMSO-pretreated control group. This inconsistency suggests that ATRA pretreatment of MSCs caused the expansion of Th17 subpopulation but inhibited the secretion activities of Th17 cells. A recent multiple center clinical trial demonstrated that anti-IL-17A monoclonal antibody secukinumab was effective for the treatment of AS [37]. Therefore, we speculate that the frequency of Th17 cells is not significantly related to the severity of AS, but the biological function of Th17 cells plays a more important role. Our results suggest that ATRA pretreatment may improve the efficacy of MSC infusion for the treatment of AS by inhibiting the secretion of IL-17A from Th17 cells.

Kezic et al. [38] demonstrated that IFN-*γ* worsened both peripheral joint and axial diseases in murine models. Abe et al. [39] found in a mouse model that upregulated IFN-*γ* was associated with ankylosing enthesitis. Zhao et al. [40] demonstrated that IFN-*γ* significantly activated the promoter of the HLA-B27 gene. IFN-*γ* is also an activator or a polarizing factor of classically activated macrophage (M1), the main source of TNF-*α* [41]. Thus IFN-*γ* is involved in the pathogenesis of AS and associated with the severity of AS. In the present study, while IFN-*γ* secreted by PBMCs was increased by DMSO-pretreated MSCs, IFN-*γ* secretion was suppressed by ATRA-pretreated MSCs at both mRNA and protein levels. Therefore, ATRA pretreatment may be able to improve the therapeutic effects of MSC infusion for the treatment of AS through downregulating IFN-*γ* production.

TNF-*α* blockade is widely used for the treatment of AS. In the present study, we observed that TNF-*α* secretion was significantly inhibited by MSCs although no such changes were found in gene transcriptions. Interestingly, ATRA pretreatment further enhanced the inhibition of TNF-*α* secretion. These results suggested MSCs, especially ATRA-pretreated MSCs, were efficient TNF-*α* inhibitors. The TNF-*α* inhibitor role of MSCs may be the major mechanism underlying the therapeutic effects of MSCs for the treatment of AS. Therefore, ATRA could be used to further improve the MSC infusion therapy for AS.

In summary, our data show that (1) ATRA pretreatment affects the immunomodulatory function of MSCs by influencing the proliferation of CD4+ T cells; (2) ATRA pretreatment on MSCs results in a reduction of AS-related pathogenic cytokines IL-17A, IFN-*γ*, and TNF-*α*; (3) a significant reduction of TNF-*α* may be the main mechanism underlying the therapeutic effects of MSC infusion in AS patients and ATRA pretreatment may further improve the efficacy; (4) IL-6 is upregulated by ATRA and environmental proinflammatory mediators, and it may act as an anti-inflammatory cytokine in AS though it leads to an expansion of Th17 subpopulation. Our findings may improve the therapeutic effects of MSC infusion for the treatment of AS. But further* in vivo* studies are needed to confirm these statements.

## Supplementary Material

Supplementary materials contains:
Supplementary Figure 1: Cytokine secretion measured by CBA under different circumstances. MSCs secreted only IL-6 but barely other cytokines. In the co-culture of MSCs and PBMCs, the production of IL-6 and IFN-γ was significantly high, especially IL-6. These seven bands stood for seven different cytokines within the detecting spectrum of the CBA kit. The fluorescence intensities were positively related to cytokine concentrations.
