# Supplementary material for: All-Trans Retinoic Acid Improves the Effects of Bone Marrow-Derived Mesenchymal Stem Cells on the Treatment of Ankylosing Spondylitis: An In Vitro Study
Source: Stem Cells Int. 2015 Jun 1;2015:484528. doi: 10.1155/2015/484528 (PMC4466433; doi:10.1155/2015/484528)
Supplement: Supplementary file 1 — Supplementary materials contains: Supplementary Figure 1: Cytokine secretion measured by CBA under different circumstances. MSCs secreted only IL-6 but barely other cytokines. In the co-culture of MSCs and PBMCs, the production of IL-6 and IFN-γ was significantly high, especially IL-6. These seven bands stood for seven different cytokines within the detecting spectrum of the CBA kit. The fluorescence intensities were positively related to cytokine concentrations. [file 484528.f1.pdf]

Supplymentary Figure and Legend

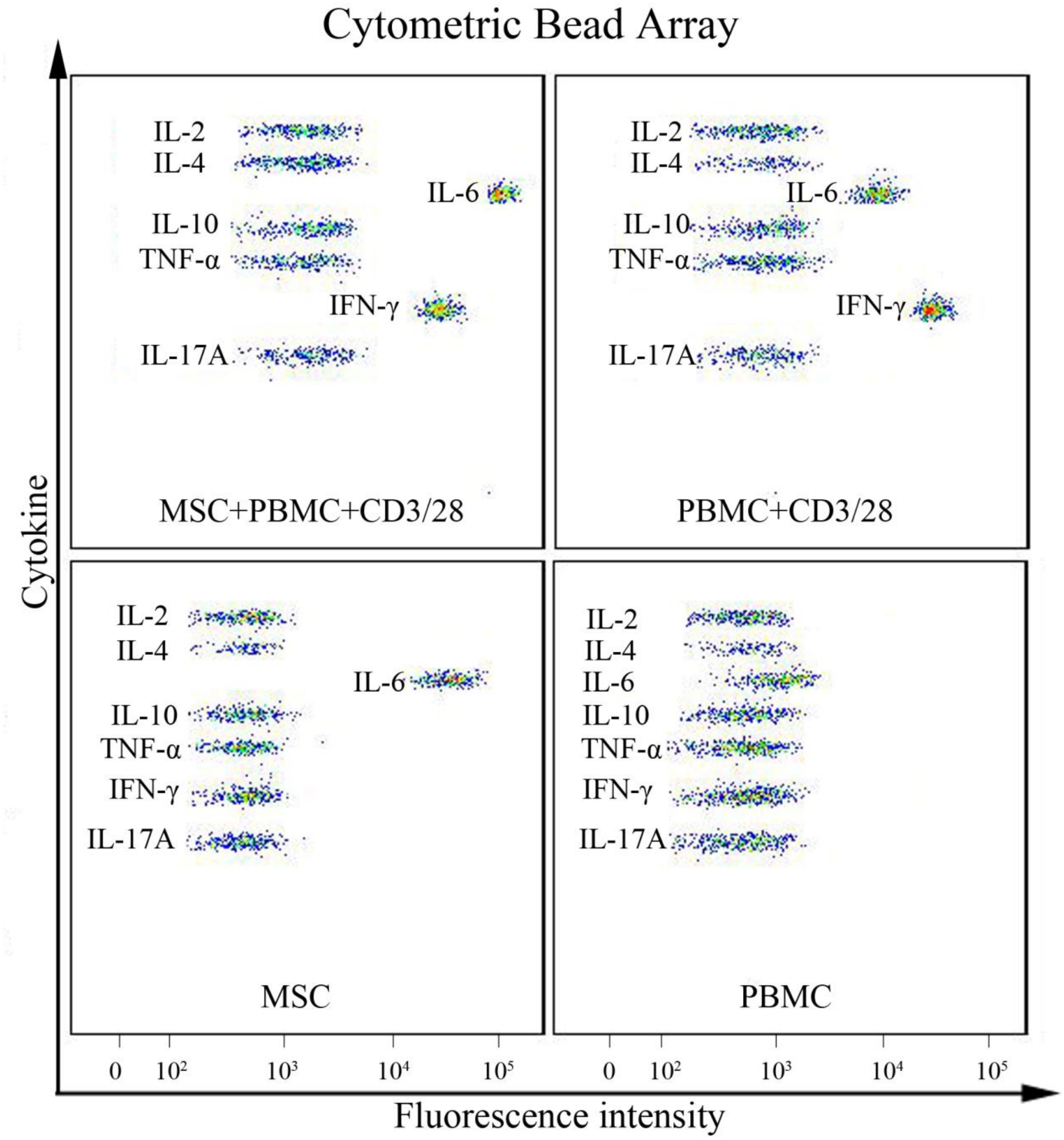

SUPPLYMENTARY FIGURE 1. Cytokine secretion measured by CBA under different circumstances. MSCs secreted only IL-6 but not other cytokines. In the co-culture of MSCs and PBMCs, the production of IL-6 and IFN-γ was significantly high, especially IL-6. These seven bands stood for seven different cytokines within the CBA detecting spectrum. The fluorescence intensities were postively related to cytokine concentrations.
